# Supplementary material for: Saccharomyces boulardii (CNCM I-745) improves intestinal damage in sepsis by remodeling intestinal flora structure
Source: Front Cell Infect Microbiol. 2025 Jul 31;15:1602792. doi: 10.3389/fcimb.2025.1602792 (PMC12350329; doi:10.3389/fcimb.2025.1602792)
Supplement: Supplementary file 1 [file DataSheet1.zip › summary/summary/7_Differential_analysis/2_LEfSe_result/Group_BW0_vs_AW0_0.05_3.0/LEfSe_Group_BW0_vs_AW0.cladogram.pdf]

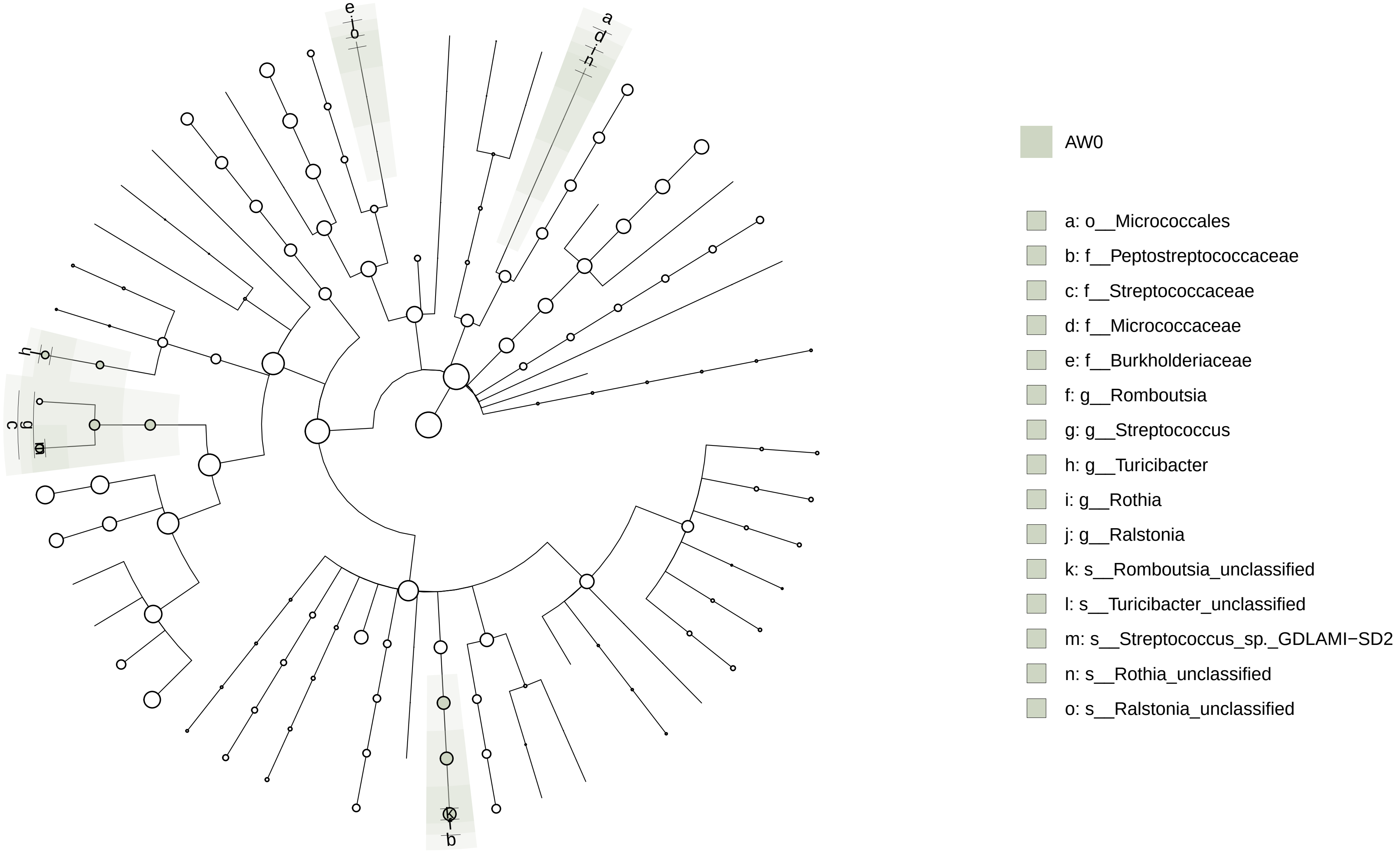

AW0
a: o__Micrococcales
b: f__Peptostreptococcaceae
c: f__Streptococcaceae
d: f__Micrococcaceae
e: f__Burkholderiaceae
f: g__Romboutsia
g: g__Streptococcus
h: g__Turicibacter
i: g__Rothia
j: g__Ralstonia
k: s__Romboutsia_unclassified
l: s__Turicibacter_unclassified
m: s__Streptococcus_sp._GDLAMI−SD2
n: s__Rothia_unclassified
o: s__Ralstonia_unclassified
